# Supplementary material for: Melanin-Based Compounds as Low-Cost Sensors for Nitroaromatics: Theoretical Insights on Molecular Interactions and Optoelectronic Responses
Source: ACS Omega. 2025 Jul 15;10(29):31908–20. doi: 10.1021/acsomega.5c03409 (PMC12311701; doi:10.1021/acsomega.5c03409)
Supplement: Supplementary file 1 [file ao5c03409_si_001.pdf]

SUPPLEMENTARY MATERIAL

Melanin-based compounds as low-cost sensors for nitroaromatics: Theoretical insights on molecular interactions and opto-electronic responses

João P. Cachaneski-Lopes [a,b], Felipe Hawthorne [c,d], Cristiano F. Woellner [c,d], Toby L. Nelson [e], Roger C. Hiorns [b], Carlos F. O. Graeff [f], Didier Bégué [b], Augusto Batagin-Neto [*,a,g]

[a] *São Paulo State University (UNESP), School of Sciences, POSMAT, Bauru/SP 17033-360, Brazil.*

[b] *CNRS/University of Pau and the Adour Region/E2S (UPPA), Institute of Analytical Sciences and Physicochemistry for the Environment and Materials, UMR5254, 64000, Pau, France.*

[c] *Federal University of Paraná (UFPR), Department of Physics, 81530-015, Curitiba/PR, Brazil*

[d] *Federal University of Paraná (UFPR), Interdisciplinary Center for Science, Technology, and Innovation (CICTI), 81530-000, Curitiba/PR, Brazil*

[e] *University of Tennessee, Oak Ridge Innovation Institute, Oak Ridge/TN, P.O. Box 2008, MS6173, USA.*

[f] *São Paulo State University (UNESP), School of Sciences, Department of Physics and Meteorology, Bauru/SP, 17033-360, Brazil.*

[g] *São Paulo State University (UNESP), Institute of Sciences and Engineering, Department of Sciences and Technology, Itapeva/SP 18409-010, Brazil.*

[*]Corresponding author: a.batagin@unesp.br

Summary

S1. Total density of states (DOS) of compounds 9a-b and NACs.. S2

S2. Adsorption methodologies.. S3

S3. Additional data for adsorbed systems (clusters obtained via CAFI)..S4

S4. Results for clusters obtained via docking by aISS.. S11

S1. Total density of states (DOS) of compounds 9a-b and NACs

Figure S1 shows the DOS of compounds 9a and 9b superimposed to the NACs. Note that the LUMO of the analytes 1,3-DNB, 2,4-DNP, 2,4-DNT, 2,6-DNT, TNP and TNT are located into the band gap of the melanin-inspired compounds.

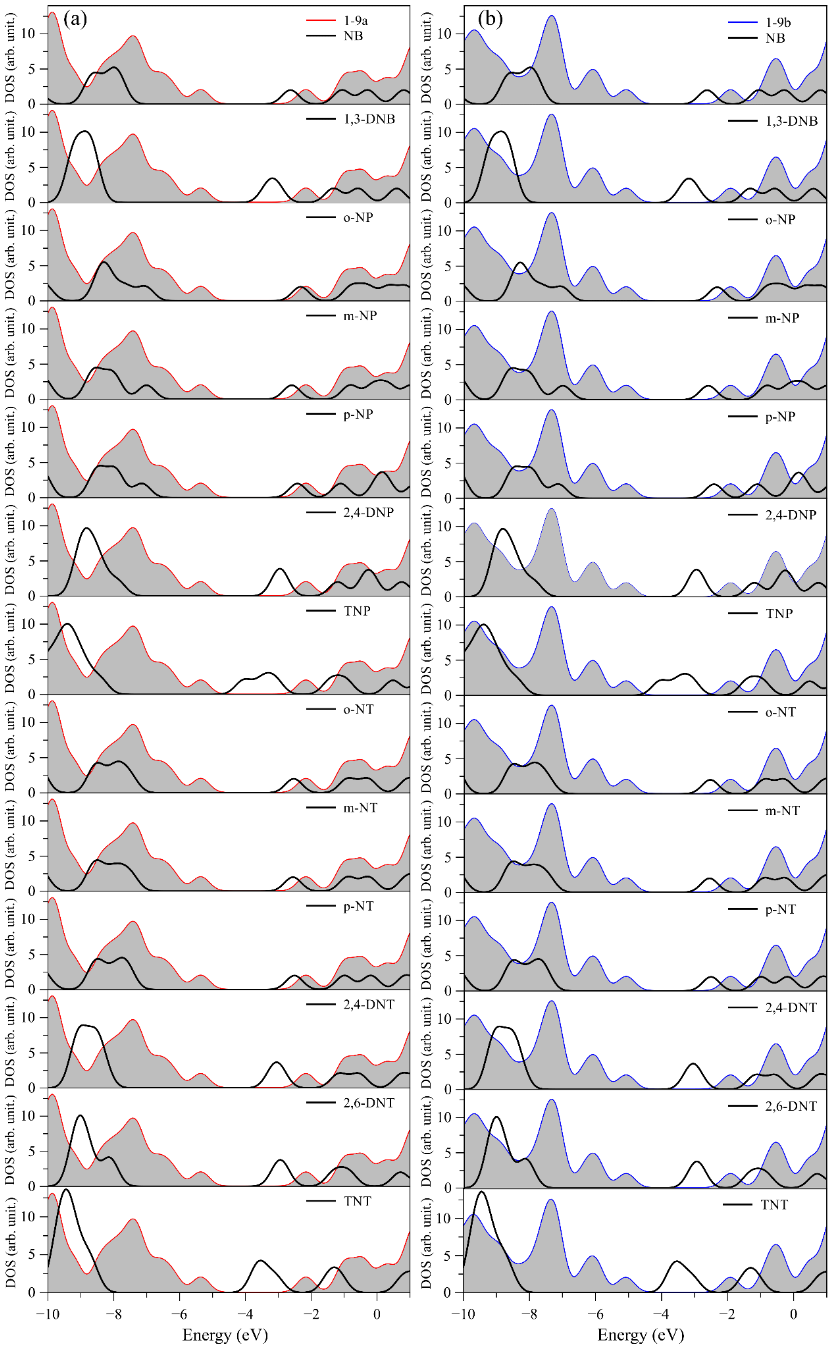

Figure S1. Comparative analyses of the DOS melanin-inspired polymers (a) 1-9a and (b) 1-9b in relation to all the Nitro-aromatics compounds.

S2. Adsorption methodologies

Two distinct adsorption processes were employed to generate substrate+analyte clusters: i) adsorption guided by CAFI and ii) Adsorption via docking submodule by automated interaction site screening (aISS). Figure S2 presents the total energy variation between each approach. Note that all variations result in positive values showing that the approach *i* leads to clusters with lower total energies.

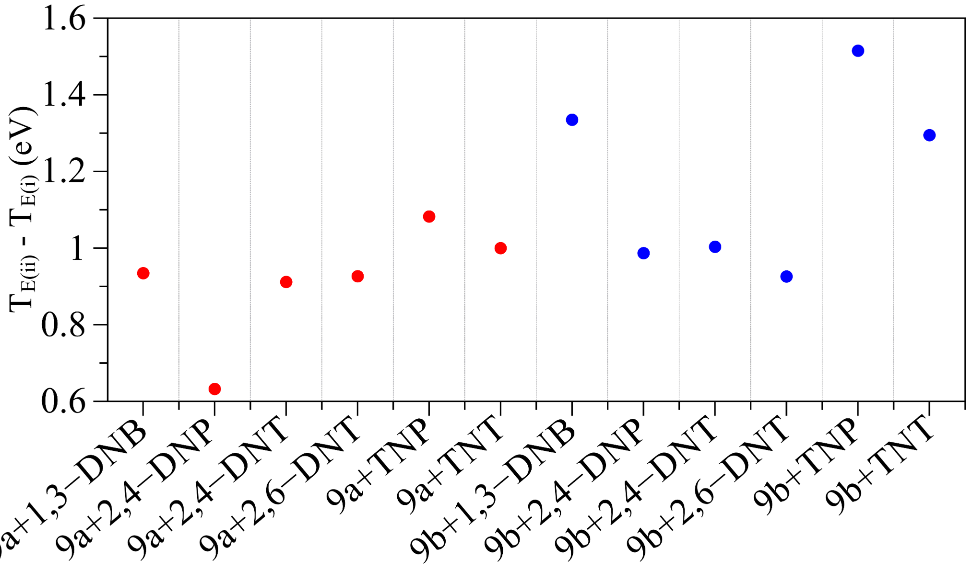

Figure S2. Comparison of total system energy of the systems obtained by the distinct adsorption approaches.

S3. Additional data for adsorbed systems (clusters obtained via CAFI)

Figures S3 and S4 show the CAFIs and MEPs of melanin-based compounds complexed with NACs.

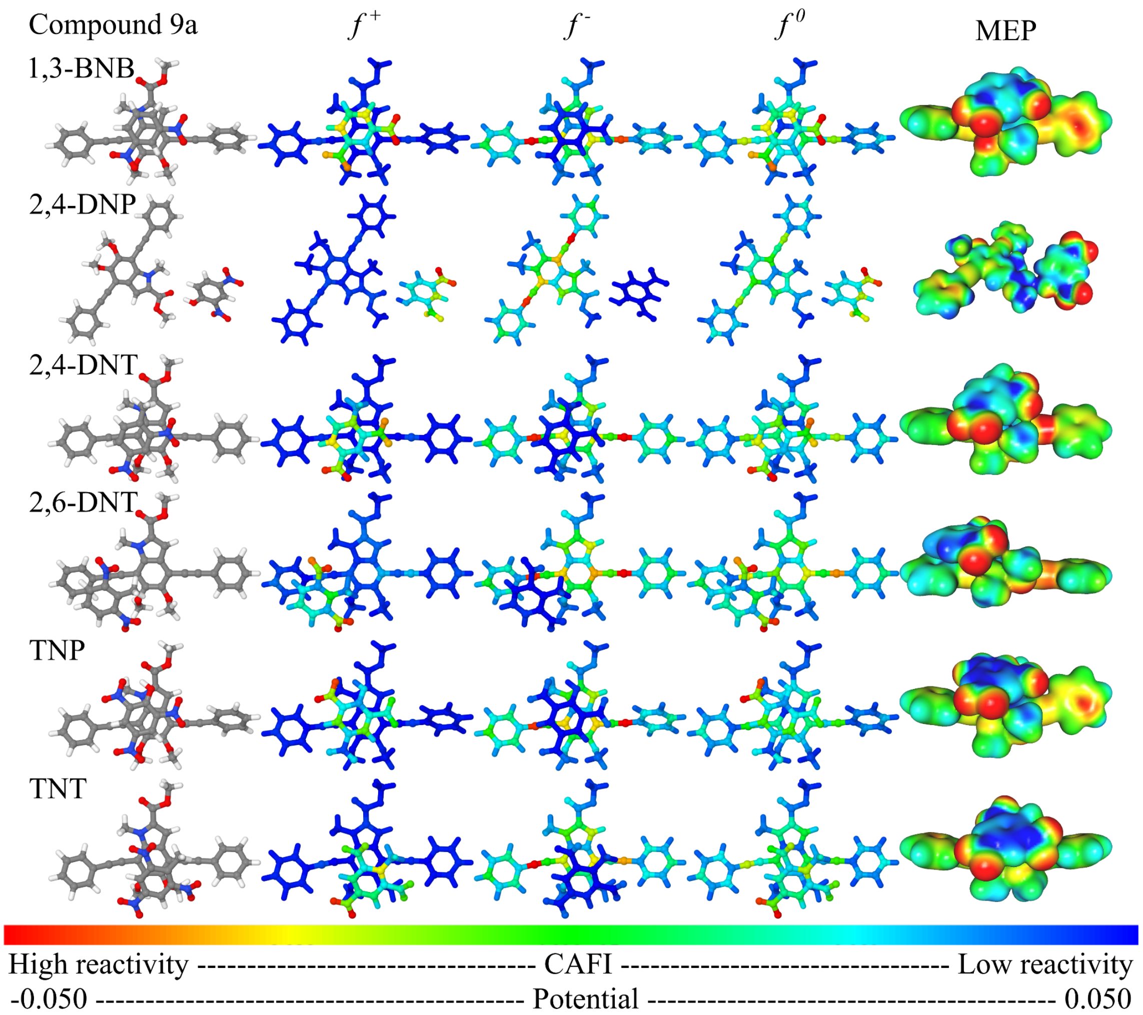

Figure S3. CAFIs and MEPs estimated for compound 9a with analytes.

Sites with higher CAFI values (red sites) represent regions that tend to interact with nucleophiles (electron acceptor sites) for f^+, electrophiles (electron donor sites) for f^- or free radicals (with no change in total number of electrons) for f^0. It is expected that the analytes will have higher f^+ indices as the LUMO of the system tends to accept electrons and that the highest f indices will be found in the melanin compounds as the HOMO tends to donate electrons, supporting the data shown in Figure 4. The CAFI of adsorbed systems show which atoms should be involved in the charge transfer processes. In this sense, special attention must be paid to the triple bonds of melanin-based compounds and the oxygen atoms of nitro groups in NACs.

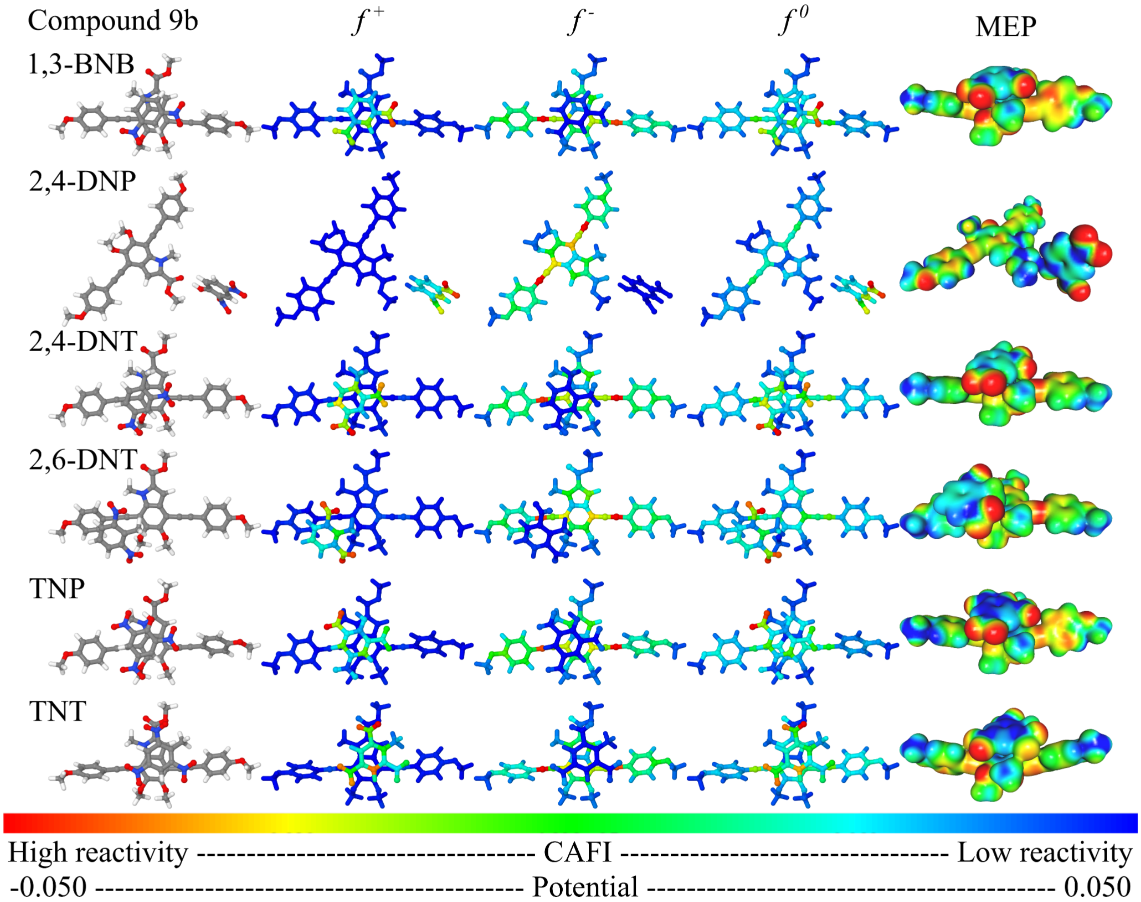

Figure S4. CAFIs and MEPs estimated for compound 9b with analytes.

Figure S5 shows the CAFIs f^+ and f^- of the melanin-based and NACs superimposed on the lowest clusters aiming to represent the proximity between the atoms with the highest reactivity indexes.

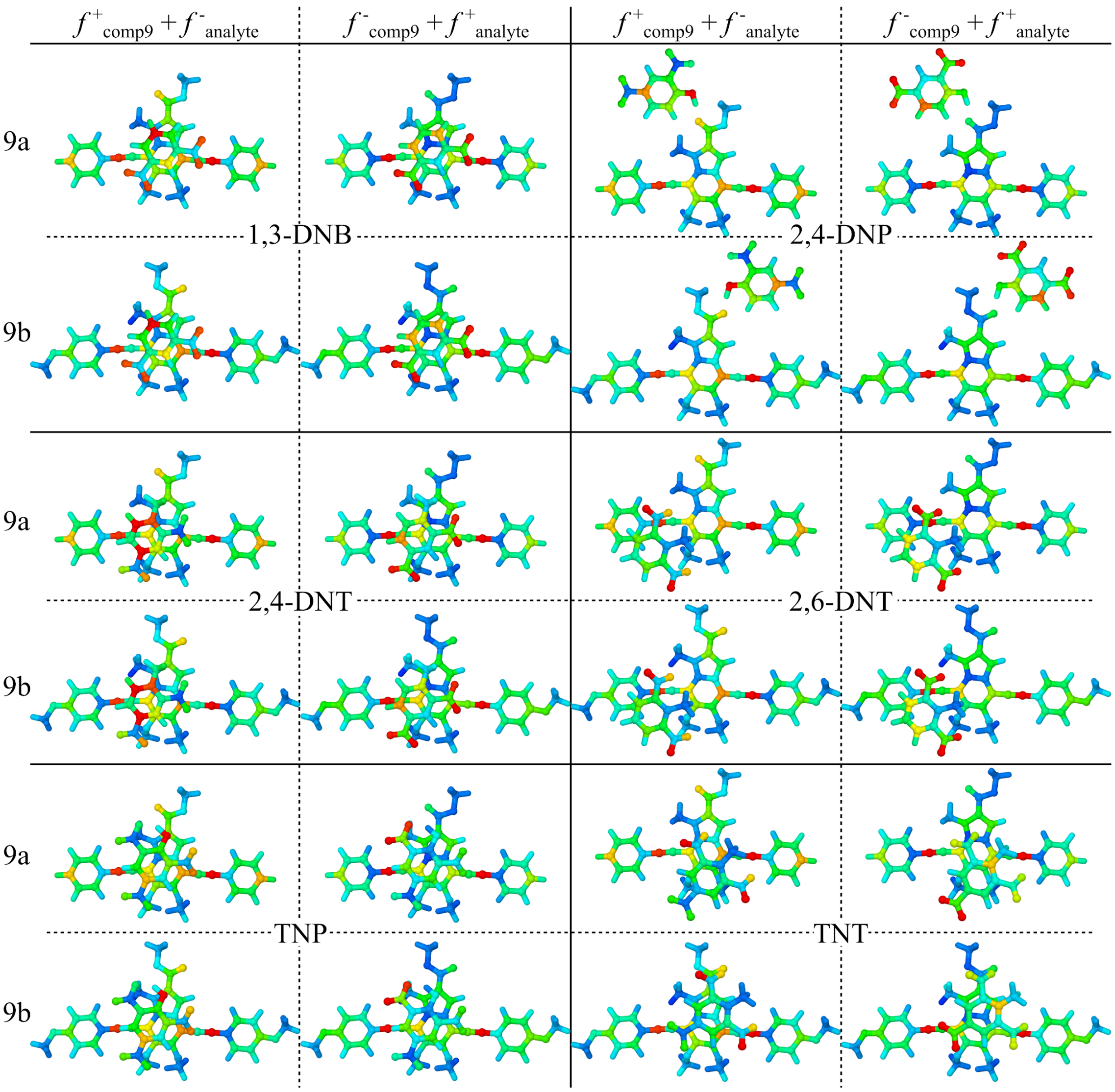

Figure S5. CAFIs estimated for compound 9a and CAFIs estimated for analytes.

Note that effective interaction between oxygen atoms of NACs' nitro groups and triple bonds of the melanin-based compounds is observed for 1,3-DNB, TNP (for compounds 9a and 9b), and TNT (for compound 9b).

Table S1 presents the theoretical optical absorption spectra of compounds 9a and 9b, both in their isolated forms and after adsorption, (clusters obtained through the CAFI approach).

Table S1. Theoretical optical absorption spectra of compounds 9a and 9b isolated and adsorbed, clusters obtained via CAFI approach.

**Compound**	**Analyte**	E_{vert} **(eV)**	λ_{max} **(nm)**	f_{osc}	**Main transit.**	ΔE **(eV)**	c_i^2 **x 100**
9a	isolated	2.9385	421.93	1.1064	**H→L**	3.19	49.2846
	1,3-DNB	3.0832	402.12	0.3962	H_3→**L**	3.71	7.9180
					$H_4 \to L_1$	4.19	5.9952
					**H**→L_2	3.43	33.3853
	2,4-DNP	2.8179	439.99	0.8466	**H→L**	2.87	48.8405
	2,4-DNT	2.9306	423.07	0.8587	**H**→L_2	3.22	48.2039
	2,6-DNT	2.9077	426.4	0.9469	**H**→L_2	3.19	48.2275
	TNP	3.0994	400.03	0.5881	H_5→**L**	3.65	1.9822
					$H_2 \to L_1$	3.45	1.4156
					**H**→L_3	3.44	43.0074
	TNT	2.931	423.01	0.7137	H_2→**L**	3.53	1.5982
					**H**→L_3	3.24	47.0102
9b	isolated	2.9032	427.06	1.3066	**H→L**	3.17	49.3717
	1,3-DNB	2.9492	420.4	0.7161	**H**→L_2	3.31	46.8609
	2,4-DNP	2.6948	460.09	0.8758	**H**→L_1	2.98	48.5907
	2,4-DNT	2.8473	435.45	0.8179	H_2→**L**	3.44	5.0127
					H_1→**L**	3.25	8.3943
					**H**→L_2	3.13	34.7204
	2,6-DNT	2.8469	435.5	0.8667	H_1→**L**	3.31	9.6336
					**H**→L_2	3.13	38.0430
	TNP	2.9776	416.39	0.6067	$H_3 \to L_1$	3.50	13.4557
					**H**→L_3	3.32	33.2629
	TNT	3.0475	406.84	0.6824	$H_3 \to L_2$	3.52	1.6672
					$H_1 \to L_2$	3.14	1.0341
					**H**→L_3	3.41	45.0711

Figure S6 shows the spatial distribution of the frontier molecular orbitals and the most relevant orbitals (largest c_i^2 x 100) presented in Table S1, resulting from the optical absorption calculations made using TD-DFT.

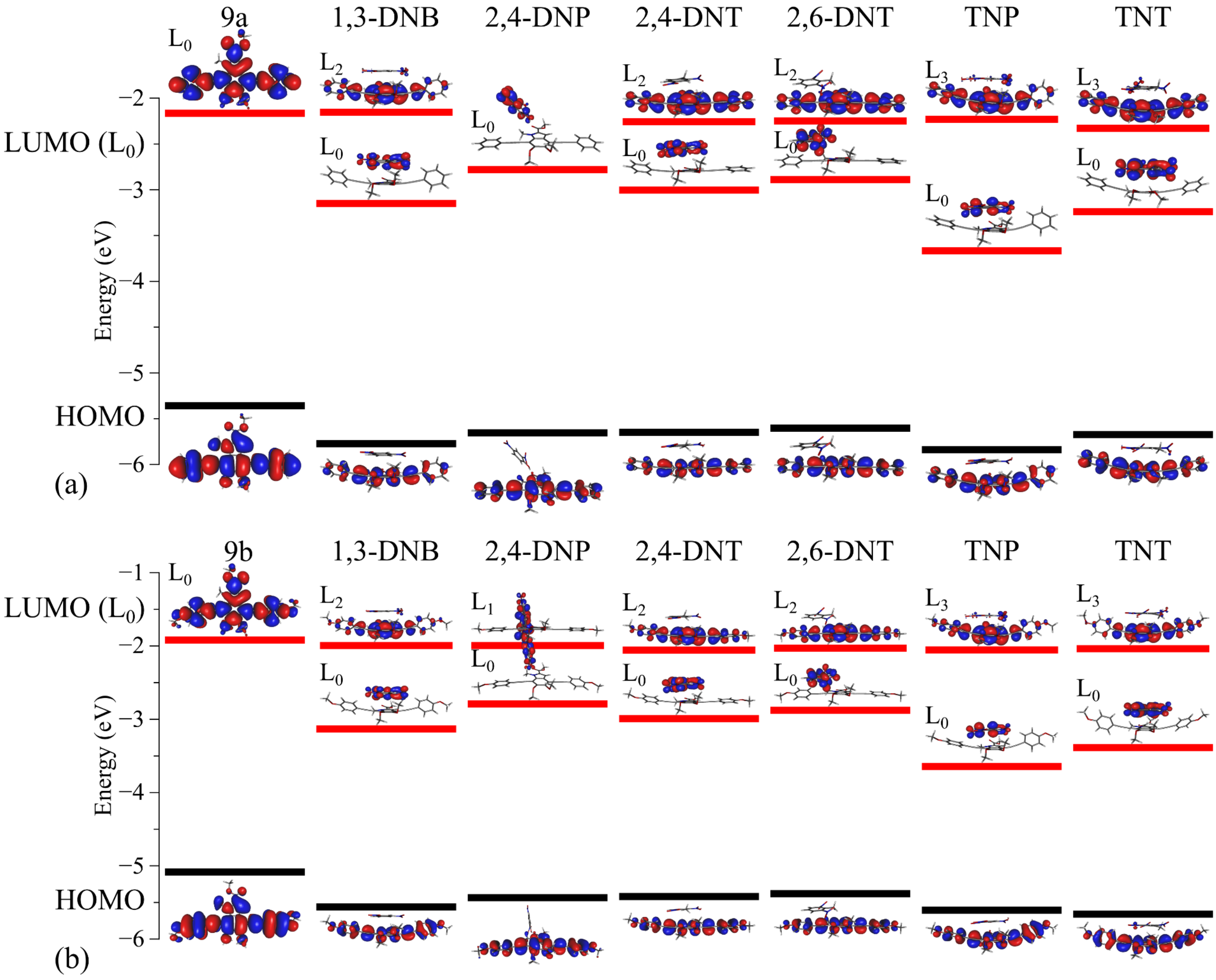

Figure S6. Spatial distribution and energy levels of the FMOs and most relevant orbitals over the clusters: (a) Compound 9a and (b) Compound 9b.

Similar to Figures 8 and 9, Figure S6 demonstrates that $LUMO_n$ (for n > 1) are centered on the melanin-based structures instead of the analytes.

The recovery time, τ, indicates the time required to completely desorb an analyte from the substrate surface. Ideally, it should range from a few milliseconds to minutes, indicating an effective anchoring property combined with a reasonable recovery capability. It can be estimated by Eq. S1:

$$\tau = \nu^{-1} \times e^{-\frac{E_{ads}}{k_B T}} \tag{S1}$$

E_{ads} refers to the adsorption energy (as presented in Fig. 11), ν denotes the attempt frequency (~10^{-12} s for visible light exposition), k_B represents the Boltzmann constant, and T represents the

temperature. Table S2 shows the τ (in seconds) estimated for TNP and TNT systems for different temperatures.

Table S2. Recovery time of compounds 9a and 9b for TNP and TNT.

Systems	τ(s)			
	**300 K**	**400 K**	**500 K**	**650 K**
9a+TNP	4.20×10^{4}	2.93	9.41×10^{-3}	4.70×10^{-5}
9a+TNT	4.41×10^{4}	3.04	9.70×10^{-3}	4.81×10^{-5}
9b+TNP	1.04×10^{5}	5.78	1.62×10^{-2}	7.14×10^{-5}
9b+TNT	1.93×10^{5}	9.21	2.35×10^{-2}	9.51×10^{-5}

To investigate the selectivity of the compounds, additional adsorption studies were carried out for N_2 and O_2 (triplet state) using the same theoretical approach as NACs presented in Table S3.

Table S3. Complexation energy of compounds 9a and 9b.

Compound	Adsorption energy (eV)							
	**1,3-DNB**	**2,4-DNP**	**2,4-DNT**	**2,6-DNT**	**TNP**	**TNT**	**N_2**	**O_2**
9a	-0.768	-0.629	-0.738	-0.669	-0.990	-0.991	-0.118	-0.102
9b	-0.784	-0.642	-0.755	-0.667	-1.013	-1.029	-0.119	-0.103

The results revealed adsorption energies of -0.118 eV (-0.119 eV) and -0.102 (-0.103 eV) for N_2 and O_2 respectively, regarding compound 9a (9b), suggesting weak interactions for both the analytes. Note that the adsorption energy obtained for N_2 (O_2) was around 8x (10x) lower than for TNP and TNT.

Figure S7 presents a quantitative description of the compounds HOMO/LUMO overlap, estimated via the spatial overlap matrix elements $\langle|\varphi_{HOMO}|||\varphi_{LUMO}|\rangle$ [1]. Note that the frontier orbitals overlap are improved for 1,3-DNB, TNP, and TNT.

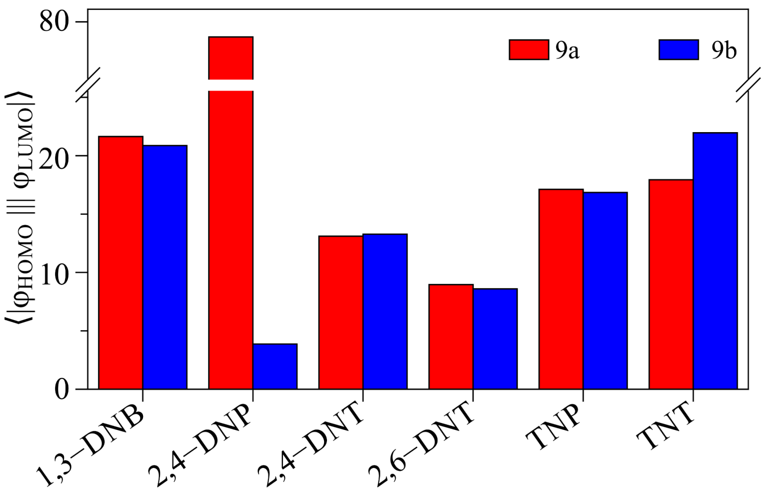

Figure S7. HOMO/LUMO spatial overlap in adsorbed systems.

S4. Results for clusters obtained via docking by aISS

Figures S8 and S9 illustrate the partial and total density of states (PDOS and DOS) obtained for clusters coming from docking by aISS (see Fig. 9 and 10 of the main text for clusters obtained by CAFI)

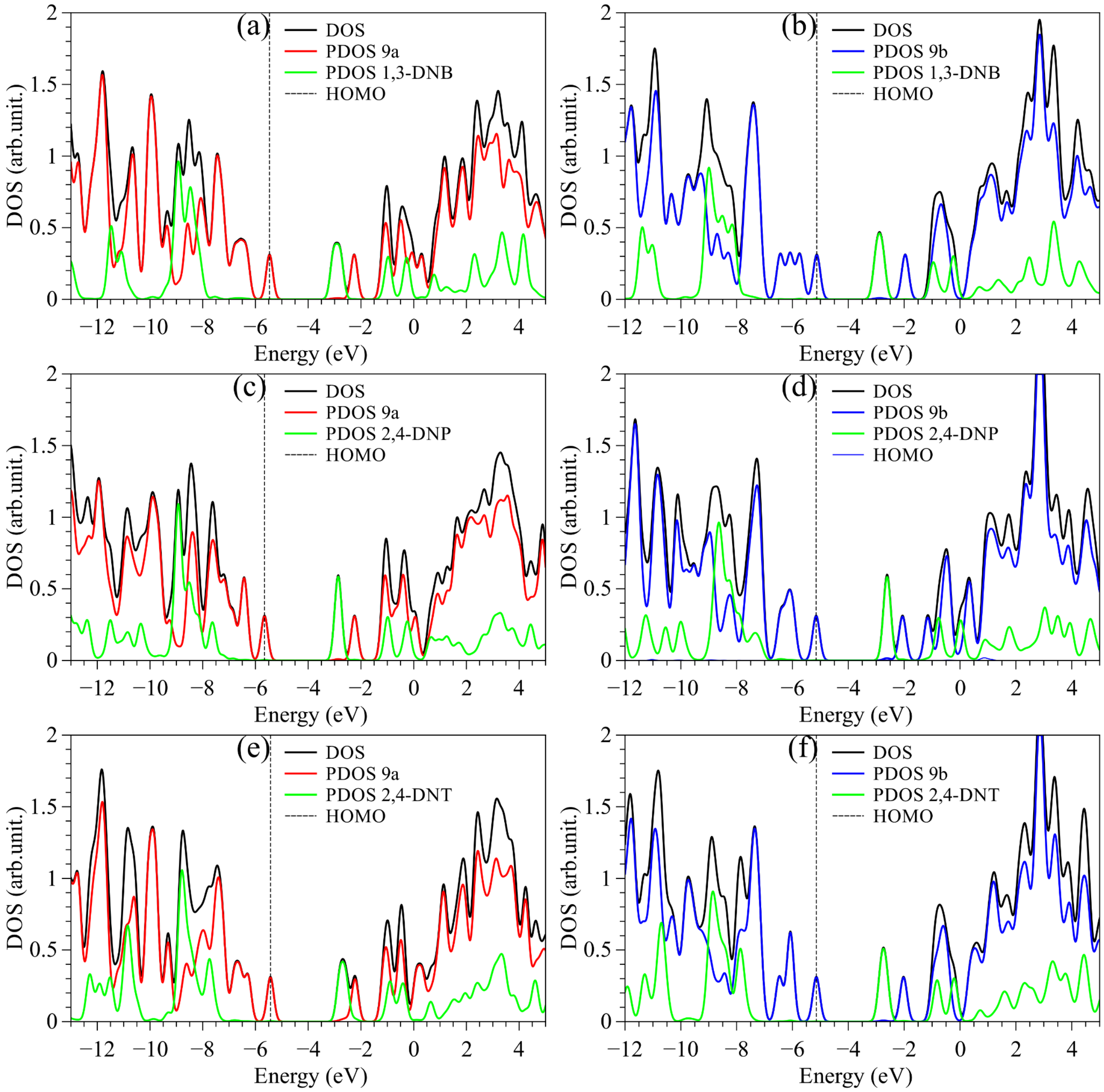

Figure S8. DOS and PDOS of melanin-inspired compound (9a - 9b) with: (a - b) 1,3-DNB, (c - d) 2,4-DNP and (e - f) 2,4-DNT positioned via docking by aISS approach.

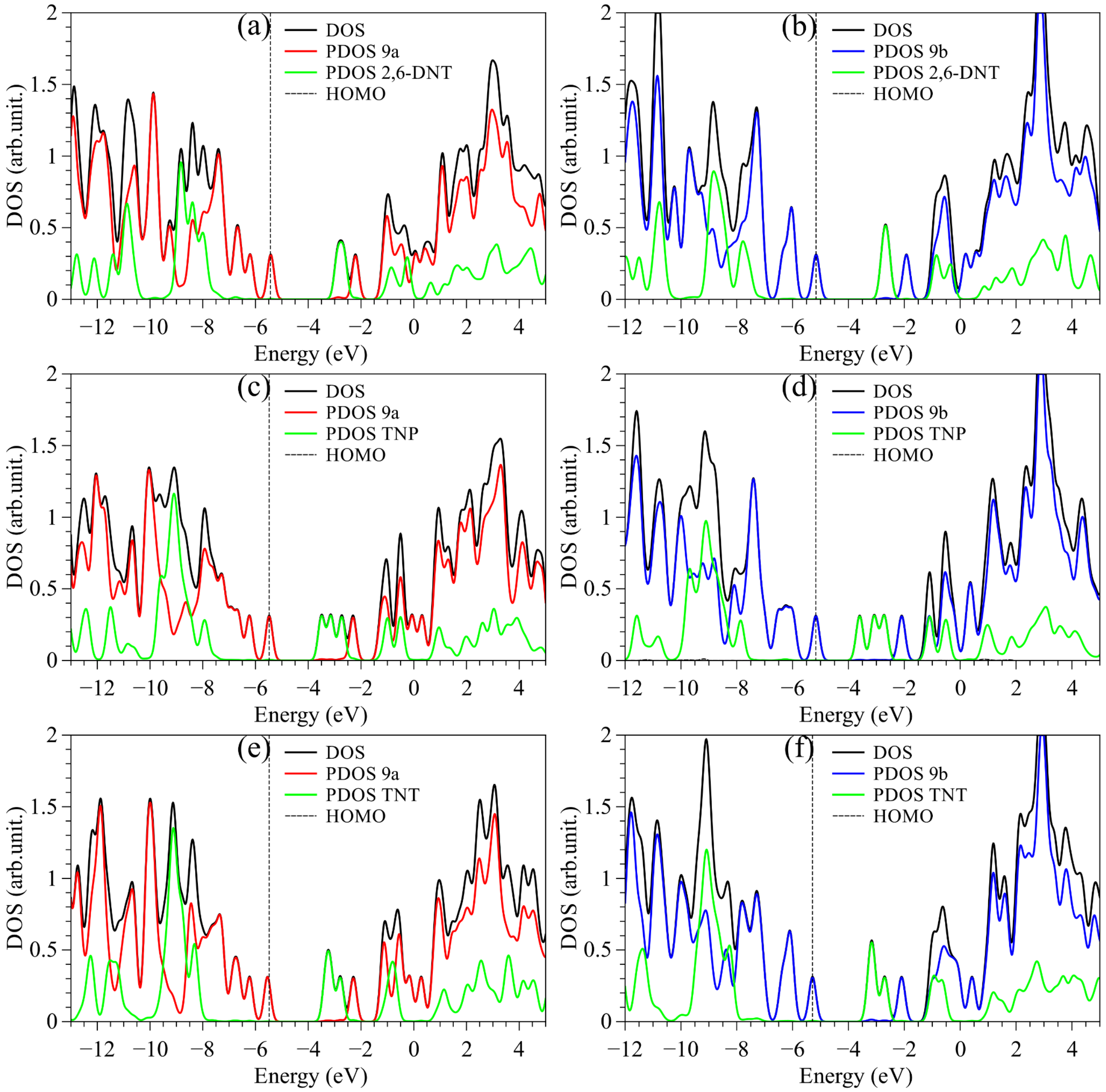

Figure S9. DOS and PDOS of melanin-inspired compound (9a - 9b) with: (a - b) 2,6-DNT, (c - d) TNP and (e - f) TNT positioned via docking by aISS approach.

Is possible to note that the HOMO of the systems is dominated by the melanin-based compounds while the LUMO is dominated by the NACs, leading to clusters with reduced electronic band gaps.

Figures S10 and S11 represent the (BSSE corrected) complexation energies between melanin substrates and analytes, and the interaction area of clusters obtained via docking by aISS approach.

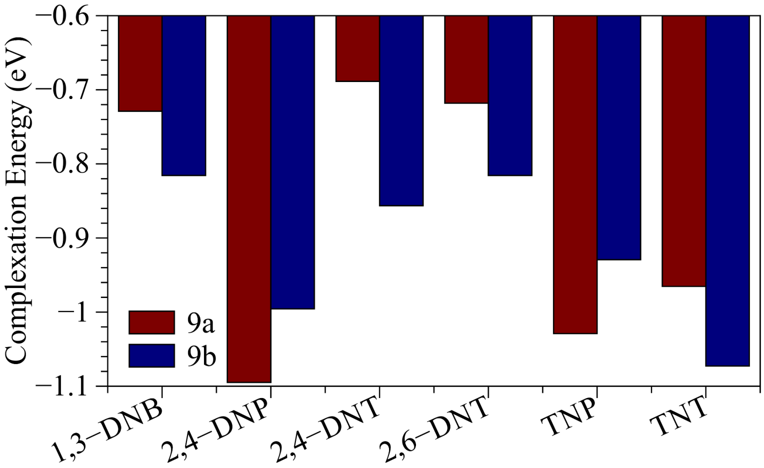

Figure S10. Complexation energies between melanin-inspired compounds and NACs for clusters obtained via docking by aISS approach.

Figure S10 shows a significant increase in the complexation energy of the clusters based on 2,4-DNP analytes. As shown, the complexation energy for 1,3-DNB, 2,4-DNT, and 2,6-DNT compounds is lower than that of the trinitro compounds. With the exception of 2,4-DNT and TNP, compound 9b exhibits a higher complexation energy compared to compound 9a.

The higher complexation energies identified for clusters obtained via docking by aISS approach indicate the existence of strong analyte-substrate interactions. However, once these systems present higher total energies in relation to CAFI guided adsorption process, we can consider that such interactions lead to more expressive structural distortions of the systems, increasing the total energy of the clusters.

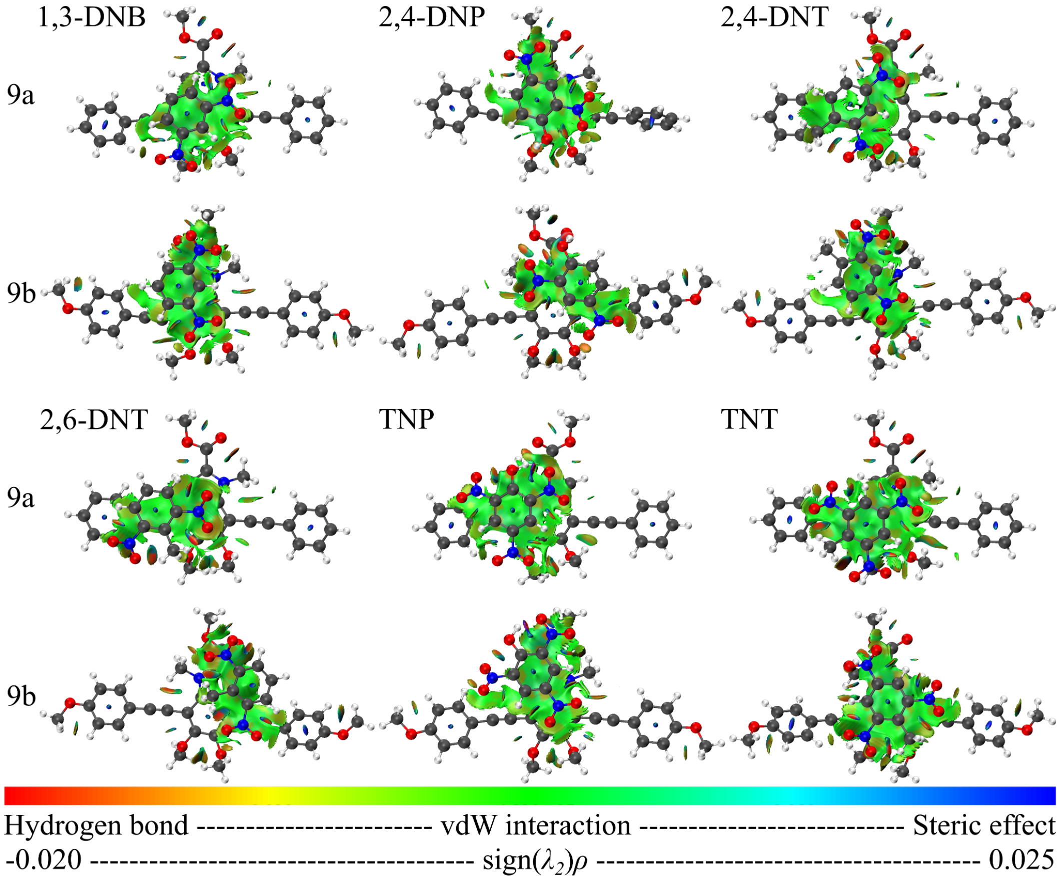

Figure S11. Analyte-melanin-based compound interactions: strength and interaction areas (results for clusters obtained via docking by aISS approach).

Note that the interactions of compound 9 with the analyte are predominantly van der Waals (vdW) interactions. Most systems exhibit interactions primarily occurring at the core of compound 9 and a portion of side groups and central rings. An exception is observed for the 9a+2,4-DNP cluster, where interactions occur with both side groups, resulting in a notable distortion of compound 9a and consequently a higher complexation energy. Except for the systems 9a-TNP, 9b-2,4-DNP, 9b-2,6-DNT, and 9b-TNT, in all the other systems the analyte final structure is aligned on the opposite side of the melanin-based compound compared to the manually assembled systems (CAFI guided adsorption).

Figure S12a-b depicts the absorption spectra of compounds 9a and 9b isolated and adsorbed with distinct NACs, as well as the main peak shift noticed for each substrate/analyte system. Figure S12c presents the numerical shift observed in Figa S12a and S12b. Figure S12d shows the variation of energy of E_{ES} (Excited state energy) of the cluster in relation to the compound 9 isolated.

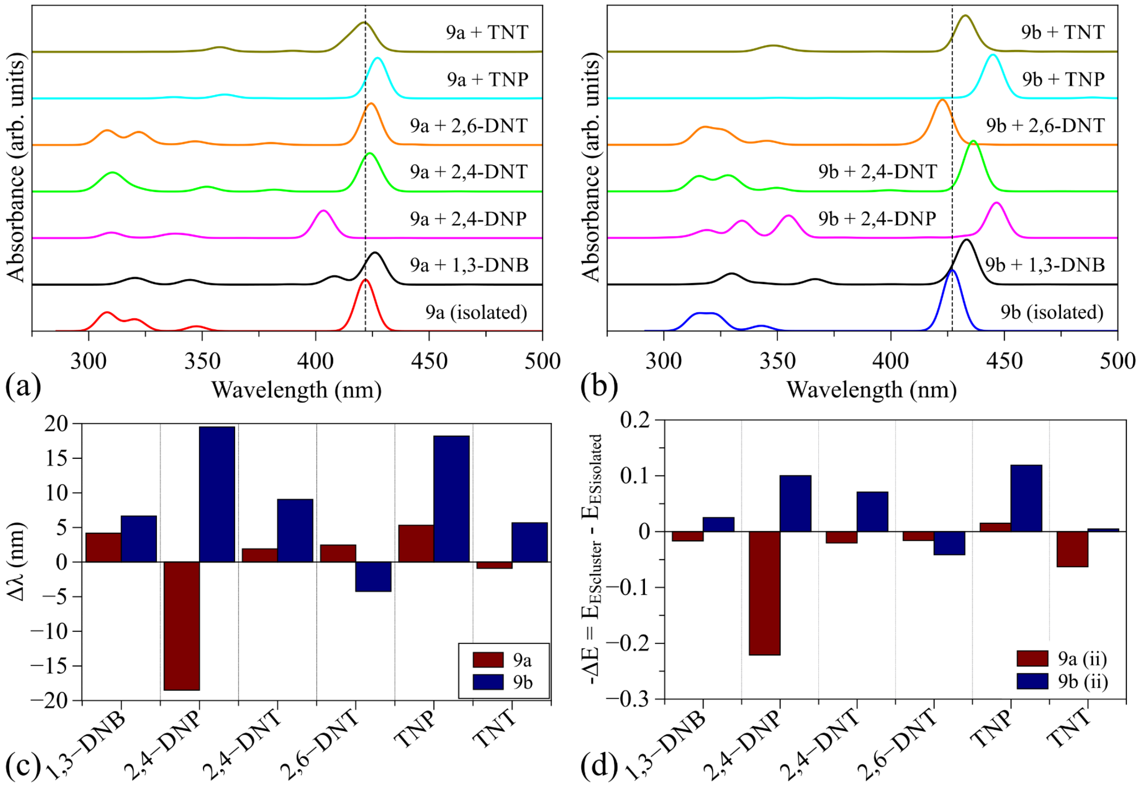

Figure S12. Theoretical optical absorption spectra of compounds 9a (a) and 9b (b) (gaussian curves with half width of 5 nm): isolated and adsorbed with NACs clusters obtained via docking by aISS approach, (c) the absorption shift and (d) Excited state energy variation.

Note that, in general, the NACs adsorption leads to more significant changes on the main peak optical absorption of the compound 9b than 9a. Some compounds presented bathochromic shift (red shifted: 1,3-DNB; 2,4-DNT; and TNP) and anomalous (with no pattern: 2,4-DNP; 2,6-DNT and TNT) analytes. Significant deviations are noticed for 2,4-DNP ($\Delta\lambda = -18.5$ nm with 9a and $\Delta\lambda = +19.5$ nm with 9b) and TNP ($\Delta\lambda = +18.18$ nm with 9b).

With the exception of 9a with 1,3-DNB, 2,4-DNT and 2,6-DNT clusters, a decrease in energy results in a red shift, while an increase in energy results in a blue shift.

Table S4 presents the theoretical optical absorption spectra of compounds 9a and 9b, both in their isolated forms and after adsorption, (clusters obtained via docking by aISS approach).

Table S4. Theoretical optical absorption spectra of compounds 9a and 9b adsorbed, clusters obtained via docking by aISS approach.

**Compound**	**Analyte**	E_{vert} **(eV)**	λ_{max} **(nm)**	f_{osc}	**Main transit.**	**ΔE (eV)**	c_i^2 **x 100**
9a	isolated	2.9385	421.93	1.1064	$\mathbf{H}\rightarrow\mathbf{L}$	3.19	49.2846
	1,3-DNB	2.9056	426.7	0.6088	$H_2\rightarrow\mathbf{L}$	3.54	5.3991
					$H_1\rightarrow L_1$	3.55	4.8748
					$\mathbf{H}\rightarrow L_2$	3.21	38.8677
	2,4-DNP	3.0733	403.42	0.5911	$\mathbf{H}\rightarrow L_2$	3.45	47.7440
	2,4-DNT	2.9322	422.84	0.6539	$H_1\rightarrow L_1$	3.55	14.8071
					$\mathbf{H}\rightarrow L_2$	3.21	33.2272
	2,6-DNT	2.9215	424.39	0.8954	$H_1\rightarrow\mathbf{L}$	3.48	1.1366
					$\mathbf{H}\rightarrow L_2$	3.21	47.4597
	TNP	2.9019	427.26	0.8716	$\mathbf{H}\rightarrow L_3$	3.18	48.1358
	TNT	2.9399	421.73	0.5192	$H_3\rightarrow\mathbf{L}$	3.53	11.2091
					$H_2\rightarrow L_1$	3.49	4.4108
					$\mathbf{H}\rightarrow L_3$	3.26	32.0560
9b	isolated	2.9032	427.06	1.3066	$\mathbf{H}\rightarrow\mathbf{L}$	3.17	49.3717
	1,3-DNB	2.8587	433.71	0.8956	$H_3\rightarrow\mathbf{L}$	3.46	4.4808
					$H_2\rightarrow L_1$	3.30	3.0457
					$\mathbf{H}\rightarrow L_2$	3.17	41.4453
	2,4-DNP	2.7764	446.56	0.7578	$\mathbf{H}\rightarrow L_2$	3.09	47.2753
	2,4-DNT	2.8430	436.11	0.9535	$H_2\rightarrow\mathbf{L}$	3.26	2.3437
					$H_1\rightarrow\mathbf{L}$	3.25	3.4708
					$\mathbf{H}\rightarrow L_2$	3.12	42.5769
	2,6-DNT	2.9323	422.83	0.9597	$H_2\rightarrow\mathbf{L}$	3.29	1.1922
					$H_1\rightarrow L_1$	3.43	1.2728
					$\mathbf{H}\rightarrow L_2$	3.24	45.6030
	TNP	2.7846	445.25	0.8910	$H_1\rightarrow L_3$	3.91	2.9801
					$\mathbf{H}\rightarrow L_3$	3.08	45.3225
	TNT	2.8652	432.73	0.7704	$H_2\rightarrow L_2$	3.42	3.9038
					$\mathbf{H}\rightarrow L_3$	3.19	43.8575

Figure S13 shows the spatial distribution of the frontier molecular orbitals and the most relevant orbitals (largest c_i^2 x 100) presented in Table S4, resulting from the optical absorption calculations made using TD-DFT.

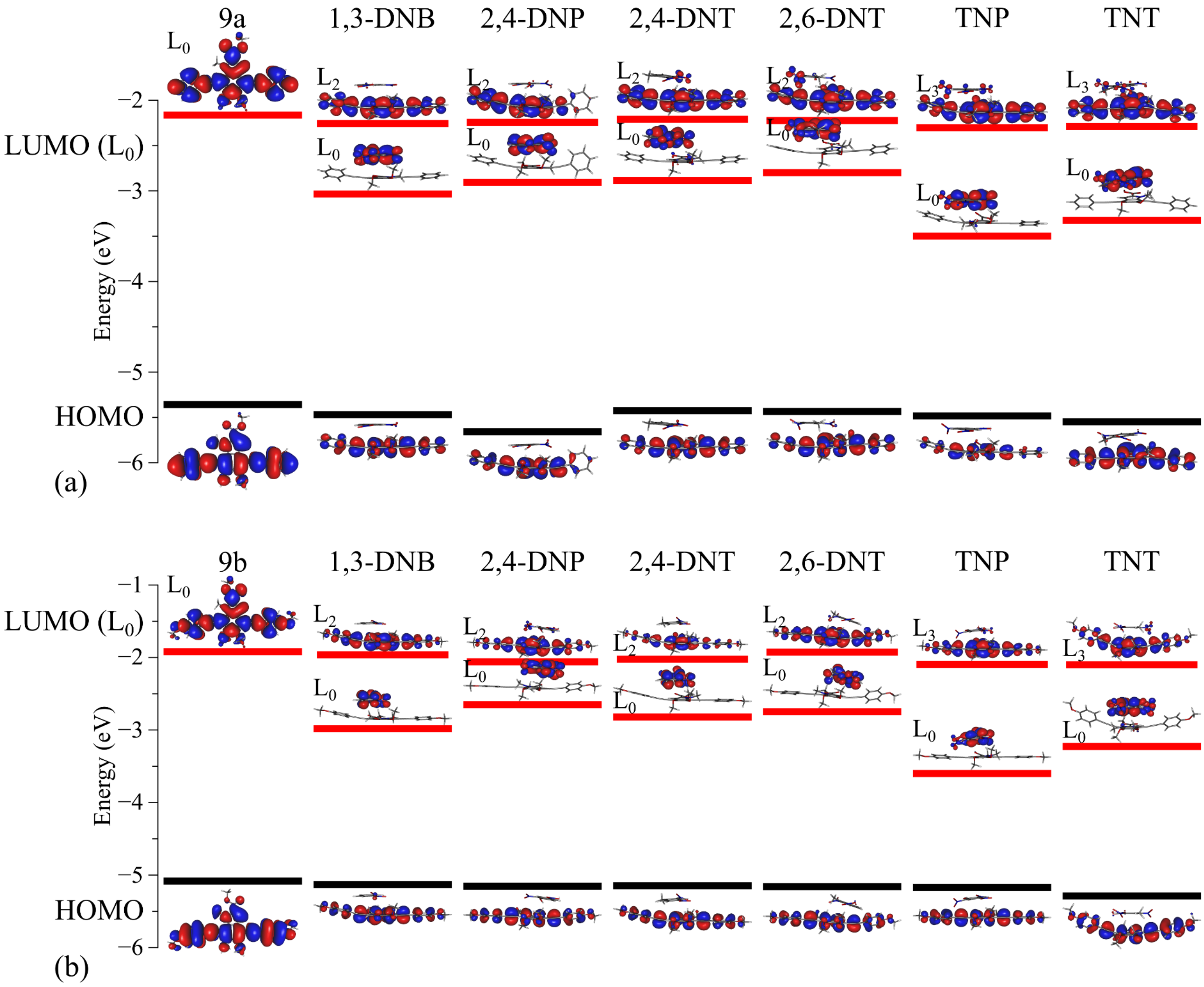

Figure S13. Spatial distribution and energy levels of the FMOs and most relevant orbitals over the clusters: (a) Compound 9a and (b) Compound 9b.

Similar to Figs S8 and S9, Figure S13 demonstrates that $LUMO_n$ (for n > 1) are centered on the melanin-based structures instead of the analytes.

References

(1) Peach, M. J. G.; Benfield, P.; Helgaker, T.; Tozer, D. J. Excitation Energies in Density Functional Theory: An Evaluation and a Diagnostic Test. *J. Chem. Phys.* **2008**, *128* (4), 044118. https://doi.org/10.1063/1.2831900.
